# LVI-PathNet: Segmentation-classification pipeline for detection of lymphovascular invasion in whole slide images of lung adenocarcinoma

**DOI:** 10.1016/j.jpi.2024.100395

**Published:** 2024-08-30

**Authors:** Anna Timakova, Vladislav Ananev, Alexey Fayzullin, Egor Zemnuhov, Egor Rumyantsev, Andrey Zharov, Nicolay Zharkov, Varvara Zotova, Elena Shchelokova, Tatiana Demura, Peter Timashev, Vladimir Makarov

**Affiliations:** a Institute for Regenerative Medicine, Sechenov First Moscow State Medical University (Sechenov University), 8-2 Trubetskaya st., Moscow 119991, Russia; b Medical Informatics Laboratory, Yaroslav-the-Wise Novgorod State University, 41 B. St. Petersburgskaya, Veliky Novgorod 173003, Russia; c Helmholtz National Medical Research Center for Eye Diseases, 14/19 Sadovaya- Chernogryazskaya, Moscow 105062, Russia; d Institute for Morphology and Digital Pathology, Sechenov First Moscow State Medical University (Sechenov University), 8-2 Trubetskaya st., Moscow 119991, Russia; e World-Class Research Center “Digital Biodesign and Personalized Healthcare”, Sechenov First Moscow State Medical University (Sechenov University), 8-2 Trubetskaya st., Moscow 119991, Russia

**Keywords:** Lung adenocarcinoma, Lymphovascular invasion, Whole slide images, Artificial intelligence, Computational pathology, Digital pathology

## Abstract

Lymphovascular invasion (LVI) in lung cancer is a significant prognostic factor that influences treatment and outcomes, yet its reliable detection is challenging due to interobserver variability. This study aims to develop a deep learning model for LVI detection using whole slide images (WSIs) and evaluate its effectiveness within a pathologist's information system. Experienced pathologists annotated blood vessels and invading tumor cells in 162 WSIs of non-mucinous lung adenocarcinoma sourced from two external and one internal datasets. Two models were trained to segment vessels and identify images with LVI features. DeepLabV3+ model achieved an Intersection-over-Union of 0.8840 and an area under the receiver operating characteristic curve (AUC-ROC) of 0.9869 in vessel segmentation. For LVI classification, the ensemble model achieved a F1-score of 0.9683 and an AUC-ROC of 0.9987. The model demonstrated robustness and was unaffected by variations in staining and image quality. The pilot study showed that pathologists' evaluation time for LVI detecting decreased by an average of 16.95%, and by 21.5% in “hard cases”. The model facilitated consistent diagnostic assessments, suggesting potential for broader applications in detecting pathological changes in blood vessels and other lung pathologies.

## Highlights

• Lymphovascular invasion in lung adenocarcinoma impacts its prognosis and treatment.
• Manual detection of invasion regions is time-consuming.
• LVI-PathNet provides segmentation of blood and lymphatic vessels.
• LVI-PathNet identifies regions of invasion inside lumens and walls of vessels.

## Graphical abstract

**Figure:**
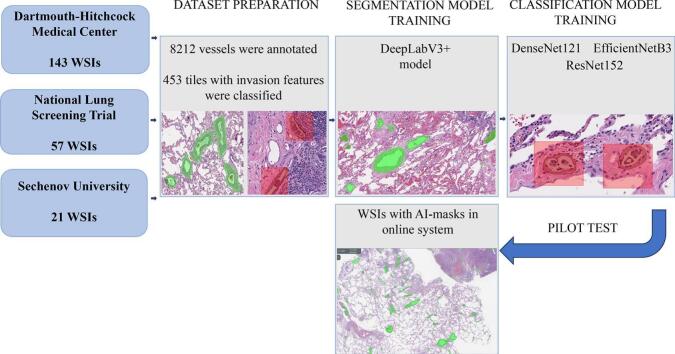

## Introduction

Lung cancer accounts for the highest mortality rate among all types of tumors globally, causing 1.79 million deaths, which is 18% of all cancer-related deaths.^1^ Differentiating tumor invasion into lymphatic and blood vessels, particularly in case of lung adenocarcinoma, is represented in all major guidelines such as CAP Cancer Protocols, TNM 8/ AJCC 8.^2^^,^^3^ Evaluating the presence of lymphovascular invasion (LVI) and microvascular density in tumor tissue is crucial for predicting metastasis and survival rates. However, concordance among pathologists regarding methodology is low, particularly concerning invasion limited to blood vessel walls. Currently, many pathologists consider such local invasion sites “easy-to-miss” and less important than the histological patterns of the cancer or its immunohistochemical and genetic status. Automating the detection of vessels with tumor invasion can bring a new perspective on the tumor growth biology and its prognostic value.

Utilizing AI and deep learning techniques demonstrates significant promise in analyzing histopathological images across various malignancies, including lung adenocarcinoma. One such method involves employing convolution neural networks (CNNs) to detect microvessels in hematoxylin and eosin (H&E)-stained images of lung adenocarcinoma tissue. Microvessel density is a prognostic factor that affects tumor development, advancement, and spread. Yi et al. pioneered a fully CNN architecture specifically designed to accurately identify microvessels within H&E images of lung adenocarcinoma.^4^ Their model, trained on a meticulously annotated dataset, demonstrated robust generalization capabilities when applied to new, previously unseen data. Subsequently, its efficacy was evaluated in whole slide images (WSIs) derived from patients within the CHCAMS cohort.^4^ During this process, segmentation masks were generated, primarily focusing on the vessel lumen, occasionally incorporating blood cells, which at times led to inaccuracies by misidentifying structures containing erythrocytes (predominantly alveoli) as vessels.

The invasion of tumor cells into lymphatic and blood vessels is another important prognostic factor in tumorigenesis, which is associated with a poor patient outcome and an increased risk of metastases in regional lymph nodes and distant sites.^3^ LVI detection is critically important for gastric cancer, in which it is often a challenging task to pathologists because it is easy to miss. Jonghyun Lee et al. proposed a deep learning-based LVI detection method which is a combination of ConViT (SMALL) patch-level classification model and YOLOX object detection model which were fine-tuned on LVI images. The classification–segmentation pipeline resulted in a heatmap of LVI regions and allowed for the object detection.^5^

In non-seminomatous germ cell tumors, presence of LVI is one of the few powerful predictors for metastasis or disease recurrence in stage I disease.^6^ In a recent study, manually annotated LVI foci were used to train a deep learning classification model to identify areas with a high prediction probability for LVI. The deep learning classifiсation model identified 34 foci, both intra- and peritumoral, in 104 slide verification images. The overall precision of the classification model in identifying areas with LVI features was 0.56. The model made false-positive identifications due to its sensitivity to artifacts.^7^

In breast cancer, pathologists evaluate lymphatic and blood vessel invasion (LBVI) separately to better assess the risk of metastasis. Manual identification of LBVI foci is time-consuming and challenging due to visual similarity to normal mammary terminal ducts, ductal carcinoma in situ, and stromal clefts. A combined DeepLabV3+ and Hover-Net deep learning model was trained to quantify morphometric features of LBVI, including both vessels and tumor cell features. The combined model identified correlations between LBVI morphometric features and risk of lymph node metastases.^8^

The problem of LVI detection in lung adenocarcinoma is still unsolved. The expected difficulties include differentiation with alveoli and the need to detect blood vessels of various calibers. Moreover, only a portion of WSIs in the datasets include LVI regions and they can be undescribed in pathology diagnoses in the clinical bases due to the low attention of the physicians.

In this study, we developed the first reported model for LVI detection in lung adenocarcinoma, which identifies regions with tumor cells in the vessel lumens of different calibers and areas of tumor ingrowth into the vessel walls.

## Materials and methods

### Dataset preparation

We used two external datasets of slide images of non-mucinous lung adenocarcinoma to train and validate the model which included 143 WSIs from Department of Pathology and Laboratory Medicine at Dartmouth-Hitchcock Medical Center (DHMC) dataset^9^ and 57 WSIs from National Lung Screening Trial (NLST) dataset.^10^ We created an internal dataset of 21 WSIs of non-mucinous lung adenocarcinoma with LVI features from the Institute of Clinical Morphology and Digital Pathology of Sechenov University (Moscow, Russia).

Slides in DHMC were scanned by an Aperio AT2 whole slide scanner at ×20 or ×40 magnification and converted to Generic tiled Pyramidal TIFF format using libvips. Slides in NLST were scanned by an Aperio AT2 whole slide scanner at ×20 or ×40 magnification and converted to Generic tiled Pyramidal TIFF format using libvips. Sechenov University slides were scanned by an Aperio AT2 whole slide scanner at ×20 magnification.

Researchers manually annotated vascular structures in QuPath v.0.4.3 and classified them into three distinct categories: (1) clean vessels without signs of LVI were annotated green; (2) vessels with invasion of epithelial displacement in the lumen; (3) vessels with intramural invasion. The classification included cases of epithelial displacement to align with the project's objective to enhance the sensitivity of the diagnostic model.

**Fig. 1:**
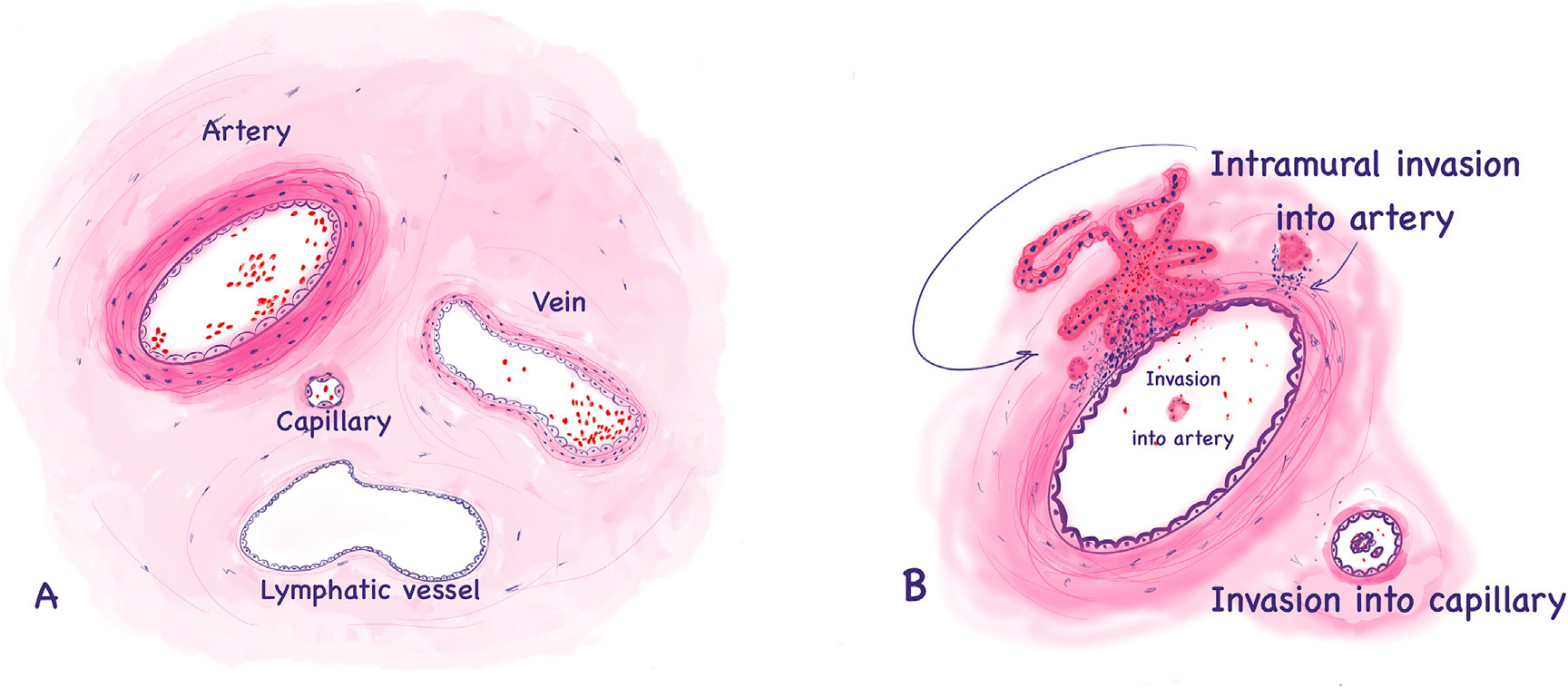
Illustrations for vascular structures in lung tissue and morphological findings of cancer cell invasion. The illustration shows the vessel types whose appearance served as a reference for the annotations (a). Reference image (b) of cancer cell invasion within the lumen and intramural invasion (cancer cells in the vessel wall).

For annotation, vessels were selected and categorized based on size and histological features into the following classes (Fig. 1):

• Small arteries and arterioles, consisting of thin tunica intima with a layer of endothelial cells on a basal membrane, prominent tunica media with smooth muscle cells and collagen fibers and tunica adventitia – a layer of connective tissue, diameter is 100–500 μm.
• Small veins and venules, consisting of tunica intima, thin or absent tunica media (especially in venules) and thin tunica adventitia, diameter is 100–200 μm.
• Capillaries, identified by a clearly visible endothelium on the basal membrane, accompanied by a pericyte layer and a loosely organized adventitial membrane, diameter is 10–50 μm;
• Lymphatic vessels are lined by a single layer of endothelial cells on a basal membrane; anchoring filaments extend from the lymphatic endothelial cells and attach to the surrounding tissue, diameter is 20–30 μm.

A significant aspect of the study involved distinguishing vascular structures from elements of the bronchial tree to avoid annotation errors. Small caliber bronchi, characterized by a single-row cubic epithelium and minimal lumen diameter, differed significantly due to their robust smooth muscle layer. Respiratory bronchioles were distinguished by a basal membrane that supported a thin, loose layer of connective tissue. Structures within the lumen that displayed free-lying layers of cylindrical epithelium or at least one ciliated cell on the basal membrane were identified as parts of the respiratory tract. This meticulous approach to annotation allowed for a clearer understanding and differentiation of vascular from non-vascular structures, enhancing the accuracy of diagnostic models used in medical research and practice.

In the process of vessel annotation, several key guidelines ensured accurate delineation and classification:

1. Vessels were annotated including all tunicae.
2. Segmentation masks outlining vessels closely adhered to the vessel's shape.
3. Vessel lumens positioned at the tissue section's edge were not subject to marking to prevent inaccuracies.
4. Segmentation mask contours exhibited smoothness, devoid of jagged edges.
5. Twisted vessels, intersected by multiple slices during preparation, were marked as separate entities, with each lumen segment receiving individual annotation.
6. Vessels intersected by the adventitia necessitated separate marking, bypassing the crossing point to avoid capturing outer sheath fibers.
7. Annotating contours of adjacent vessels did not overlap or intersect. Sacrificing marking areas in intersecting regions ensured clear demarcation.

Additionally, structures whose classification could not be reliably determined were omitted from the annotation process. Both lymphatic and blood capillaries were included in the annotation process. The lymphatic capillaries were identified by a layer of overlapping endothelial cells on a discontinuous basal membrane. These guidelines collectively ensured accurate vessel annotation.

The histological assessment of lung cancer cell invasion into blood and lymphatic vessels was based on specific criteria to distinguish between normal (clean, non-invasive) vessels, vessels with true invasion, and vessels with intramural invasion.

Invasion was defined by the presence of tumor cells within the lumen of a vessel, forming a tumor embolus. Histological signs confirming invasion included: (1) identification of erythrocytes or other blood components within the embolus; (2) detection of fibrin deposition accompanied the tumor embolus; (3) the shape of the embolus matched the vessel lumen, particularly in vessels with a smaller diameter.

The vessels with intramural invasion were characterized by the presence of cancer cells inside the vessel wall, but not within the lumen. In such vessels, tumor cells were located along the contour of the tunica adventitia. Tumor cells found within the tunica media and tunica adventitia of the vessel wall or beneath the endothelial layer were often accompanied by inflammatory infiltrates.

For the training of the classification model, the images of annotated vessels were cropped and classified into normal tiles (without cancer cells), tiles with true invasion with tumor emboli, and tiles with intramural invasion containing images of vessel walls with tumor cells.

For segmentation training and validation, Pathologist №1 (3 years of experience) manually annotated 3808 blood vessels on 105 WSIs from DHMC, Pathologist №2 (3 years of experience) manually annotated 4404 vessels on 57 WSIs from NLST. When combined and reviewed by an experienced Pathologist №3 with 10 years of experience, the dataset included 162 fully annotated WSIs containing 8212 vessels of all types. Fig. S1 of the supplementary material contains information about the process of expert validation.

For the classification model training, we used 69 WSIs (187 annotated vessels) from DHMC and 6 WSIs (29 annotated vessels) from the Sechenov University dataset which contained regions of LVI. The images of annotated vessels were cropped and only tiles with LVI features were selected, 493 tiles in total (128 × 128 pixels). Additionally, 1029 tiles of normal stroma tissue were added to the dataset. Table S1 of the supplementary material contains clinical metadata of WSI.

### Preprocessing techniques

**Fig. 2:**
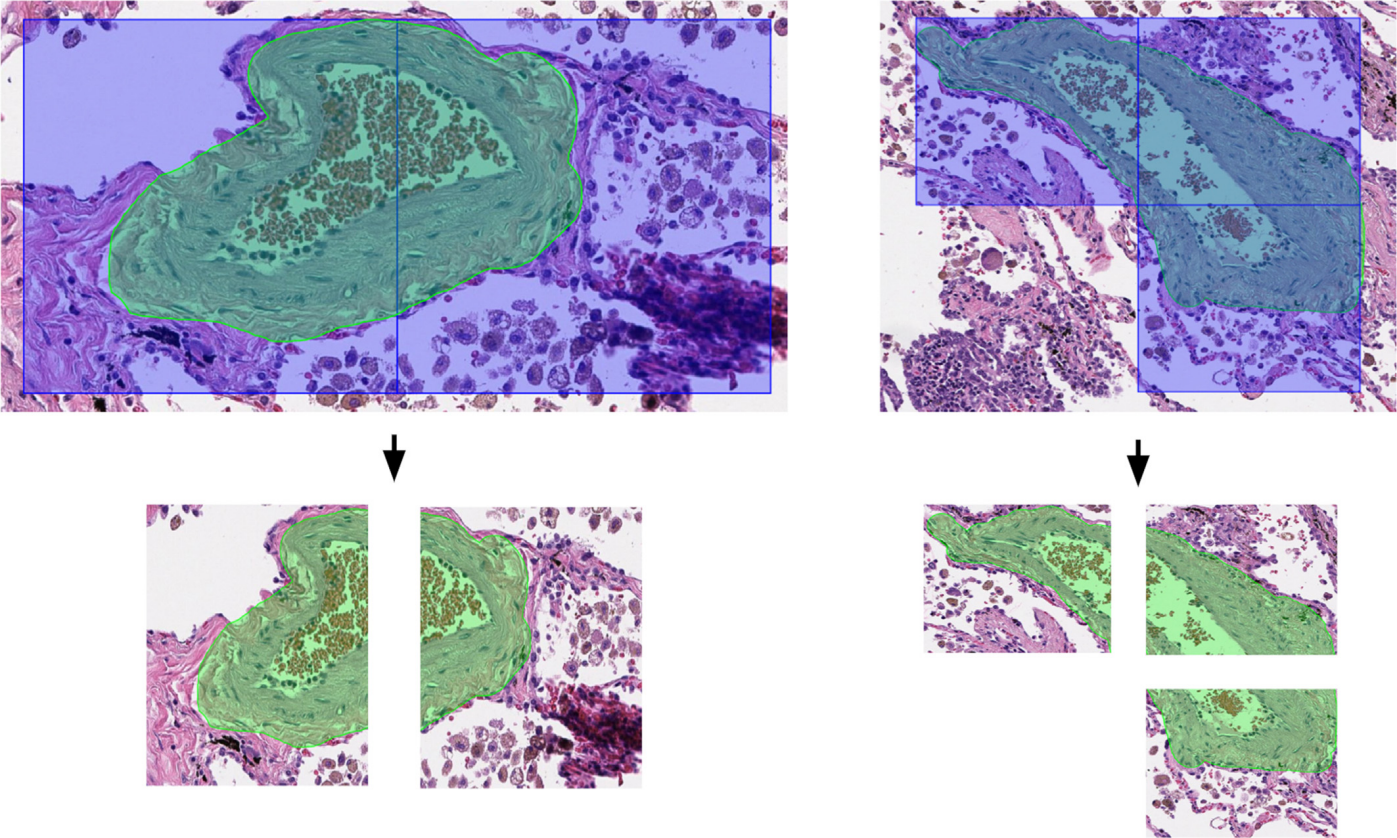
Examples of annotated blood vessel in normal lung tissue located at the edges of the tiles with side size of 128 × 128 μm.

The annotated images were processed to address a problem when tiles contained only a part of an entire vessel structure (Fig. 2). The incomplete shape of vessels could negatively impact the accuracy of a semantic segmentation model. To overcome this limitation, a padding technique based on the reflection method was employed during model training.^11^

**Fig. 3:**
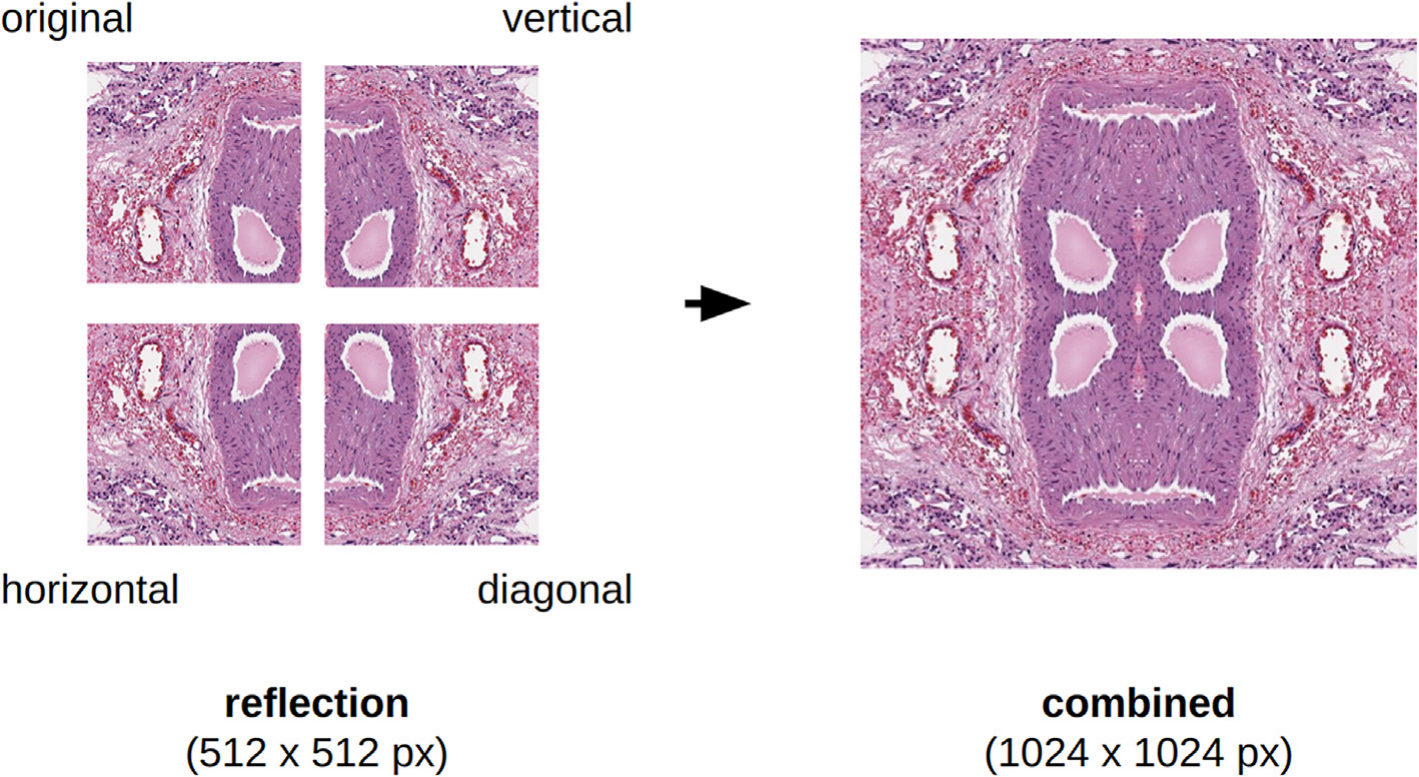
Example of reflection technique application applied to a tile containing an artery surrounded by alveolar tissue of the lung. Side size of tile – 512 × 512 pixels and 1024 × 1024 pixels.

The reflection method was applied to the original WSI tiles to reflect them horizontally, vertically and diagonally. The resulting tiles were combined with the original tile (512 × 512 pixels), resulting in an image of size 1024 × 1024 pixels (Fig. 3). The application of this padding method helped to mitigate the distortion effect in segmenting object boundaries, especially in situations where the object was located at the edge of the tile and spatial information was missing in the convolutional layers**.**

In our study, we utilized the RandStainNA method for normalizing the staining of WSIs.^12^ It was required because staining variations in histological slides often occur depending on the laboratory, timing, and conditions of the analysis. This factor can significantly affect the accuracy of machine learning models, as they may interpret these color variations as significant features, leading to overfitting, and reduced performance. RandStainNA applied random stain normalization to the training images, standardizing the color distribution across the entire dataset. This normalization technique helped models to focus on structural features rather than color variations, thereby enhancing their robustness and accuracy.

**Fig. 4:**
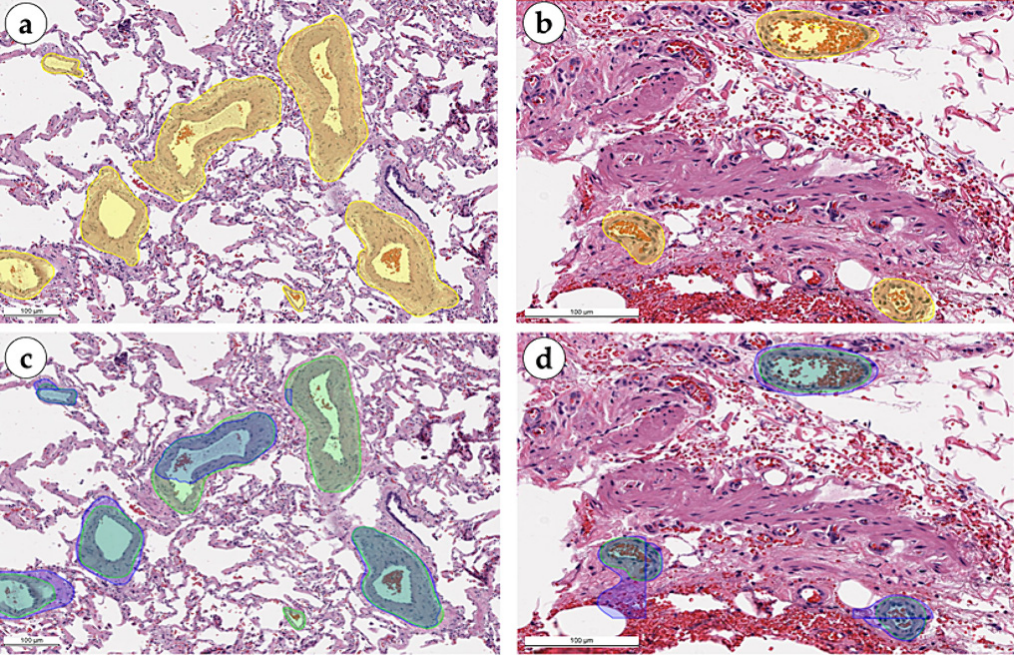
The effect of Test Time Augmentation (TTA) on vessel segmentation in whole slide images of lung tissue. Fig. 4(a), (b) represent ground truth – manual annotation by pathologist. The blue line represents the predicted mask without TTA, whereas the green line represents the predicted mask with TTA. Fig. 4(c) shows an example of under-segmentation, whereas Fig. 4(d) illustrates the tile stitching effects. Scale bar – 100 μm. (For interpretation of the references to color in this figure legend, the reader is referred to the web version of this article.)

To enhance the performance of the proposed models, regardless of their architecture, various augmentation methods were applied during model training: Flip, Rotate, Grid Distortion, Optical Distortion, Gaussian Blur, and Gaussian Noise. We also applied the Test Time Augmentation (TTA) approach to mitigate data variations or minor changes in the images by applying various transformations to the test images and subsequently averaging the results, which improved the model's final prediction^13^ (Fig. 4).

Combination data augmentation techniques such as Flip, Rotate, Grid Distortion, Optical Distortion, Gaussian Blur, and Gaussian Noise resulted in improved segmentation performance:

• FBeta: 0.941 - > 0.955
• IoU: 0.878 - > 0.8840.

There was also a visible reduction in the number of distortions in the segmentation of vessel boundaries (Fig. S2 of the supplementary material).

It should be noted that the efficacy of each augmentation method was not evaluated separately, as the focus was on the combined effect of their application.

The application of normalization and augmentation techniques allowed for the artificial expansion of the training dataset, which in turn reduced overfitting and increased the diversity of the original data representation, making the model more robust when working with data from different domains. The performance improvement effect of applying normalization and augmentations was significant to the effectiveness of the segmentation–classification pipeline due to the limited number of images in the train set, especially for the task of classification of LVI-positive regions.^14^

### Segmentation–classification pipeline training

**Fig. 5:**
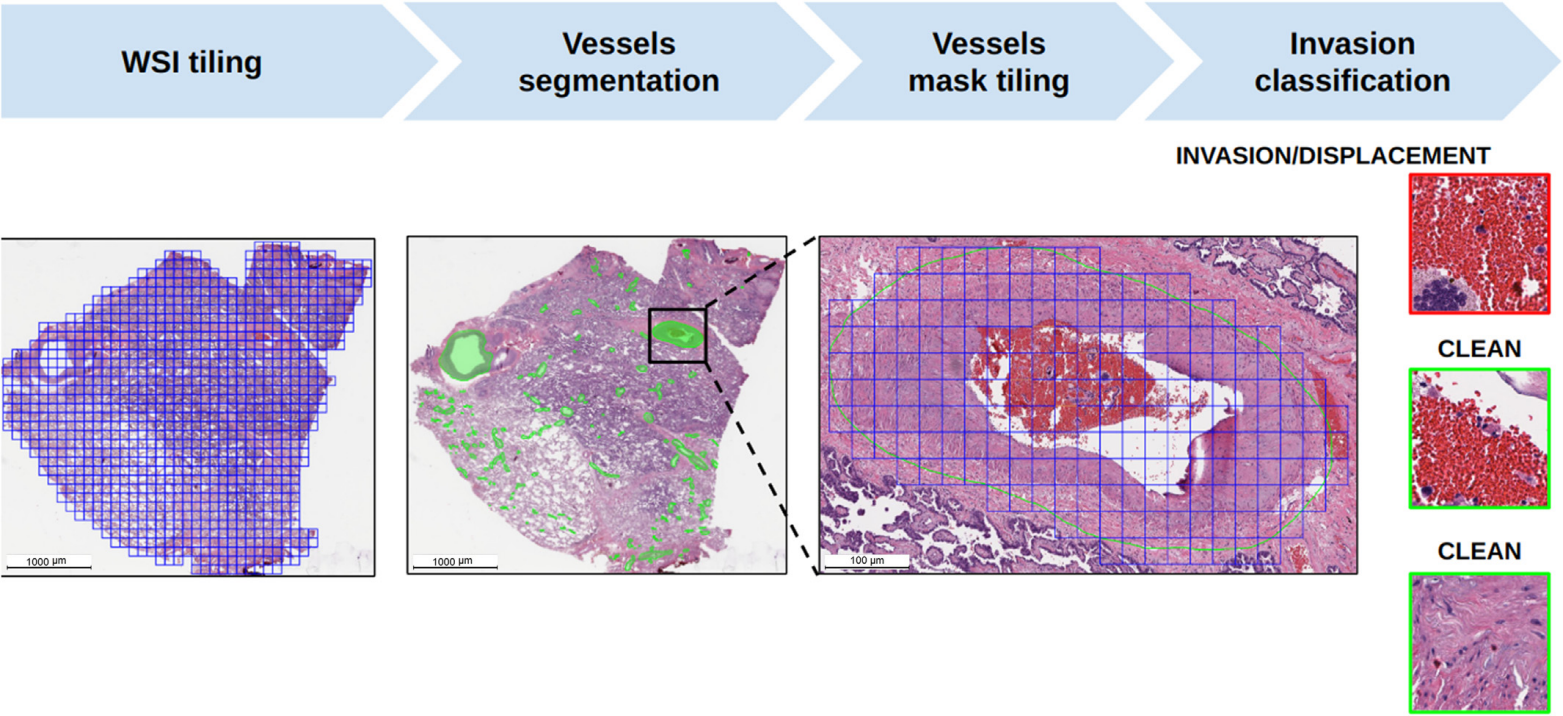
LVI-PathNet framework. The framework included the following main stages: slicing the WSI into tiles, segmenting the vessels, slicing the vessel masks into tiles, and classifying the tiles as invasive or non-invasive. Scale bar – 1000 μm.

This study proposed a framework for detecting LVI in histopathological images (Fig. 5), consisting of two models, each performing a specific task.

#### Vessel segmentation

**Fig. 6:**
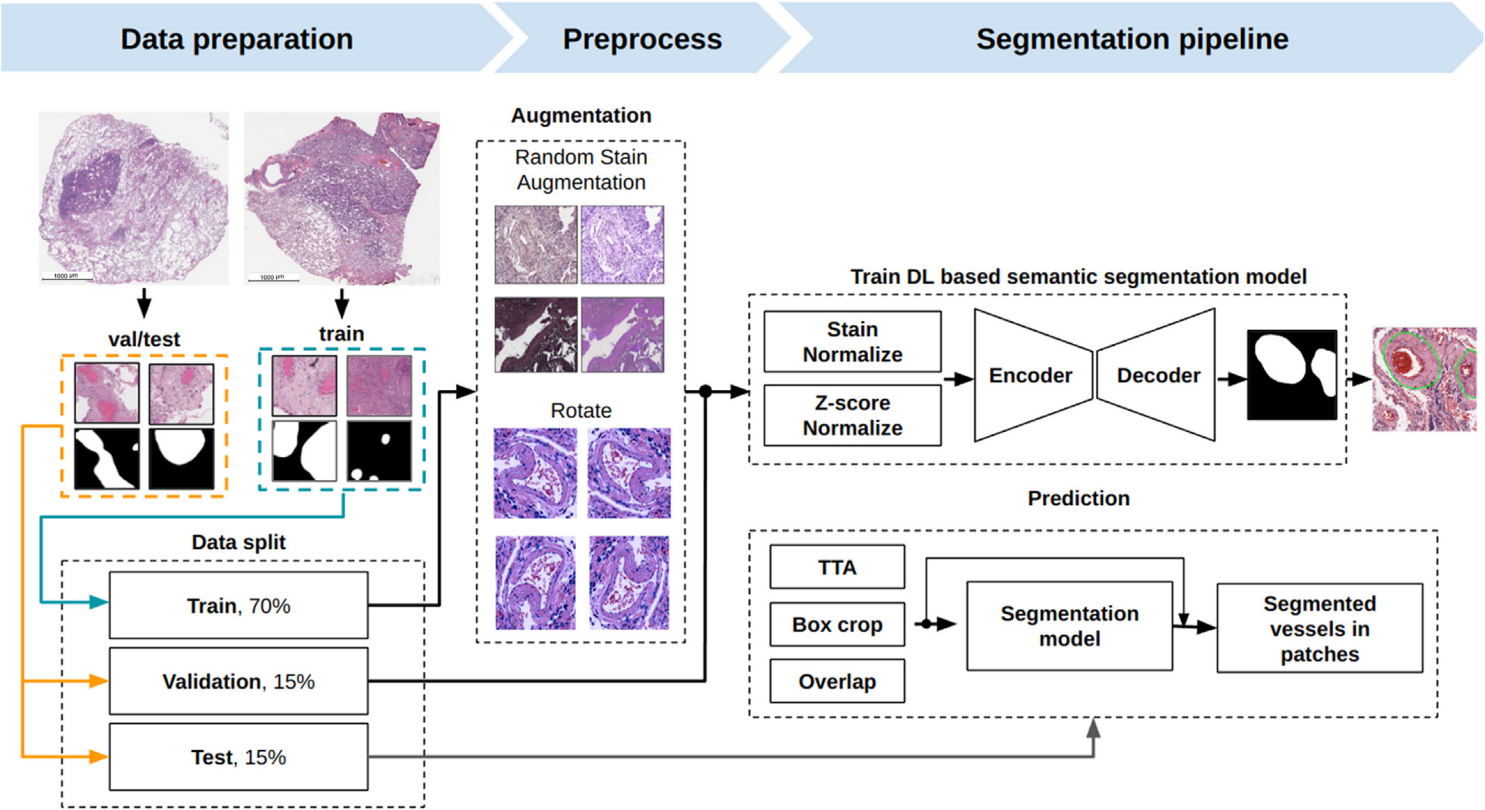
Data preparation and training of the model. The dataset was split into three parts: 70% of WSIs were sliced into tiles, stain-normalized, and rotated. These data were used to train an encoder–decoder neural network model for blood vessel segmentation. 15% of WSIs were used for validation and 15% for testing. During prediction, techniques like TTA, box cropping, and overlapping enhanced segmentation accuracy. The skip connection between the output of operations such as TTA, Box Crop, and Overlap and the predicted result of the segmentation model indicates the need to store information about the transformations applied to the original image. This information allows to perform inverse transformations of the predicted vessel masks, according to the transformations performed on the original image. The inverse transformations allow to correctly perform the averaging procedure of the predictions for each image, which ultimately improves the quality of the final segmentation mask. This approach helps to combine the results of several augmentations, minimizing errors, and improving model accuracy. The final output showed segmented vessels in tile-wise segmentation masks. Scale bar – 1000 μm.

At this stage, an encoder–decoder neural network model was employed to perform semantic segmentation of blood and lymphatic vessels in WSIs. The sequence of steps involved in vessel segmentation were shown in Fig. 6.

#### Localization of invasion regions

**Fig. 7:**
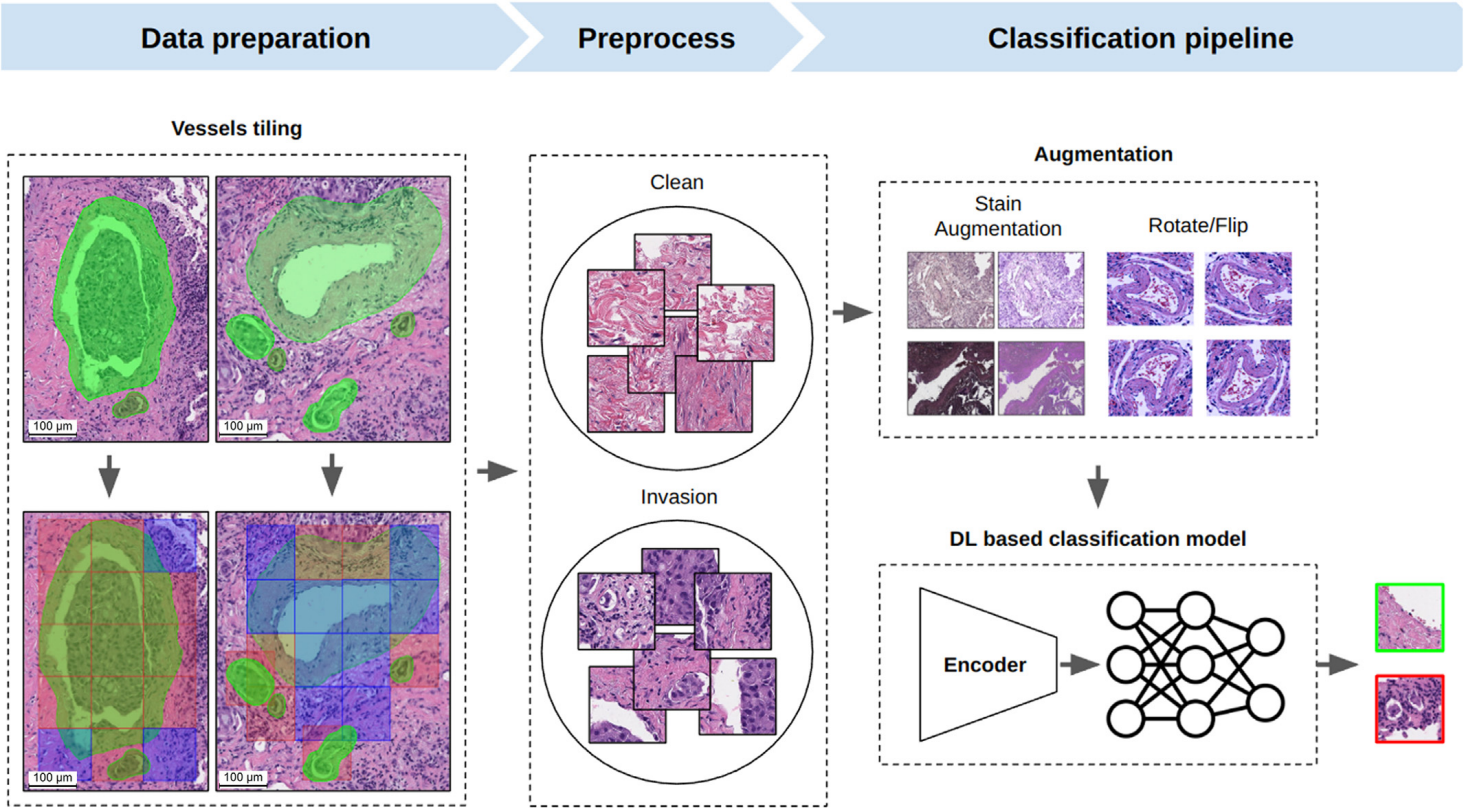
Pipeline for classifying invasive regions in vascular structures. Vessels were segmented and sliced into 128 × 128 μm tiles using segmentation masks. The tiles were divided into clean and invasive classes. Augmentations, including stain variations and rotations/flips, were applied. These augmented tiles were processed by an encoder–classifier neural network, which classified them as invasive/displacement or clean (non-invasive). Data were divided into 80% for training, 15% for validation, and 5% for testing, ensuring thorough model training and evaluation. Scale bar – 100 μm.

The detected vessels were divided into 128 × 128 μm tiles. An encoder–classifier neural network was then applied to classify these tiles into invasion/displacement and clean (non-invasive) classes (Fig. 7).

The models focused on the complex morphology and significant size variability of the vessels. Significant challenge of the study was that only 216 of 8212 annotated vessels in the WSIs exhibited LVI features. This made us to develop a framework consisting of the following main stages: slicing the WSI into tiles, vessel segmentation, slicing vessel masks into tiles, and classifying the tiles into invasive and non-invasive classes.

#### Segmentation model pipeline

**Fig. 8:**
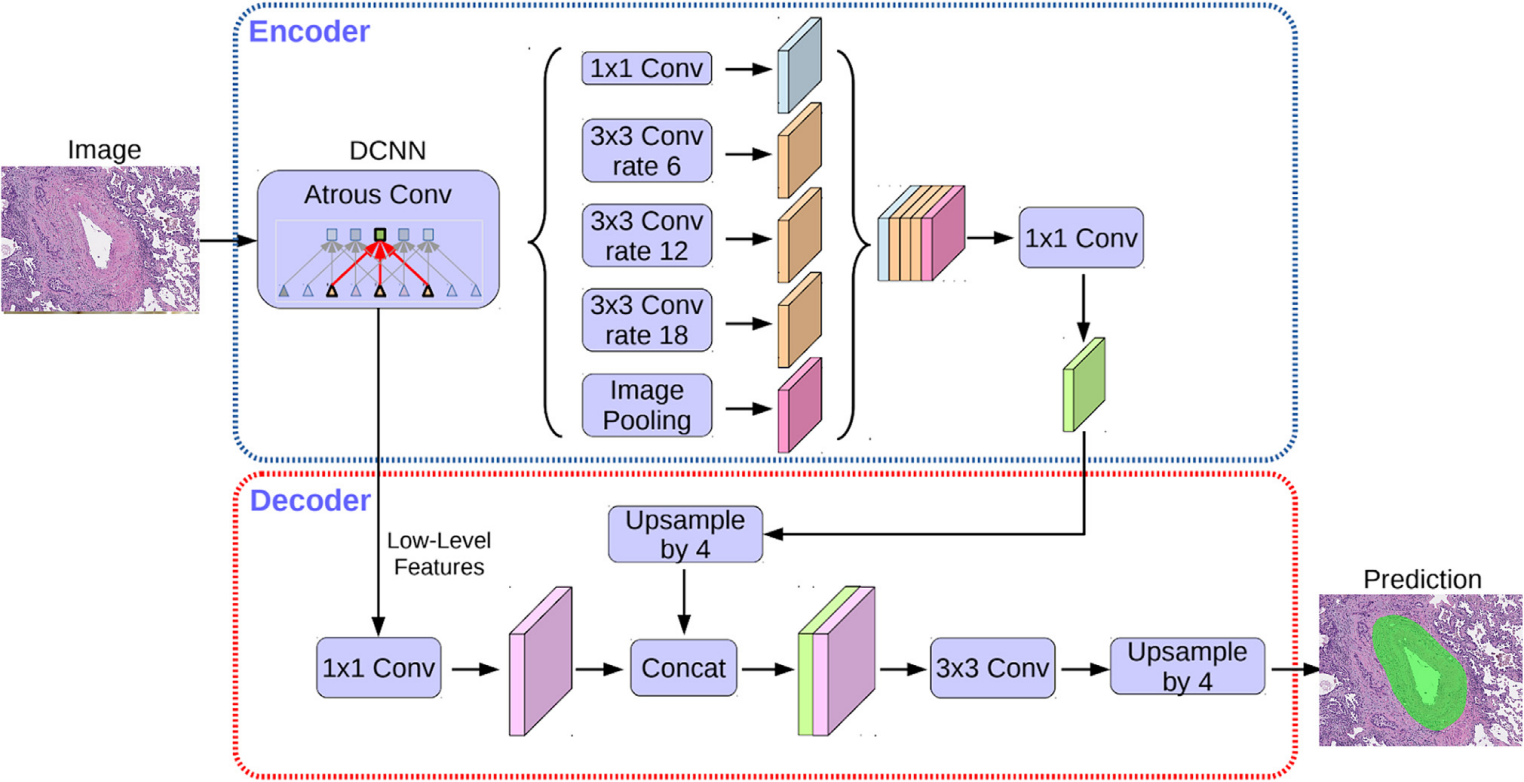
DeepLabV3+ architecture. The encoder module encodes multi-scale contextual information by applying atrous convolution at multiple scales, whereas the simple yet effective decoder module refines the segmentation results along object boundaries.^17^

For vessel segmentation in WSI, the DeepLabV3+ model was utilized. DeepLabV3+ is an advanced neural network architecture designed for semantic image segmentation.^15^ The DeepLabV3+ model with its several architectural innovations was more suitable for the task of blood and lymphatic vessel segmentation (Fig. 8) due to its advantages,^15^^,^^16^ compared to UNet, UNet++, FPN, and PSPNet alternative models. The use of atrous (dilated) convolutions enabled the model to expand the receptive field without losing resolution, capturing contextual information and details at various scales. The ASPP module in DeepLabV3+ aggregated information from multiple scales simultaneously, which was critical for accurately segmenting vessels of different calibers. The enhanced decoder ensured more precise recovery of spatial details, improving the delineation of vessel boundaries. Furthermore, the model exhibited high versatility, flexibility, stability, and robustness to data variations such as changes in staining and texture, which are common in medical images.

**Fig. 9:**
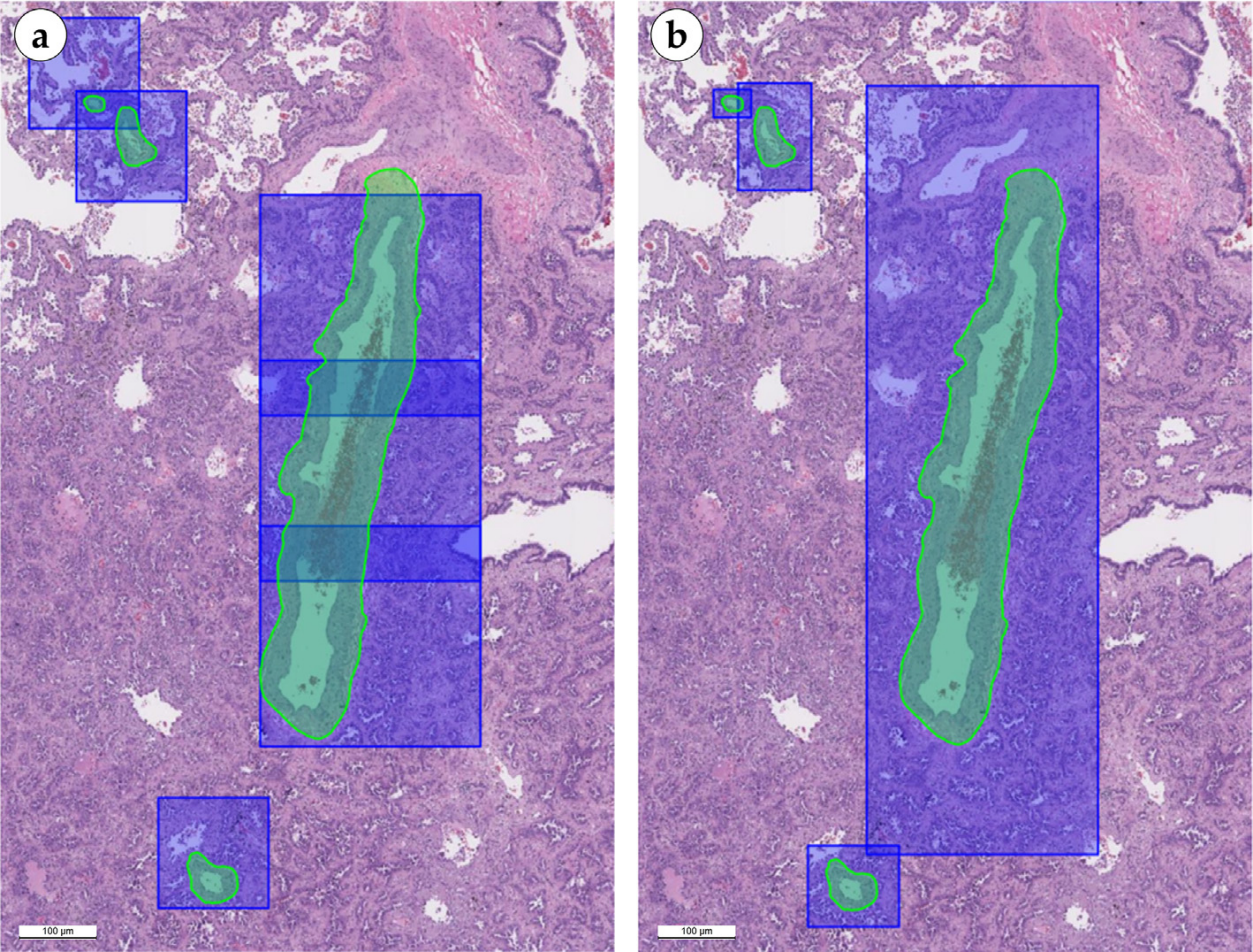
Two variants of WSI tiling for vascular segmentation are presented. Mask tiling, in which singular tiles were used for small caliber vessels, however, an elongated section of artery required several overlapping tiles to be selected (a). Bbox tiling, in which one bounding box was generated for each blood vessel (b). Scale bar – 100 μm.
• Bbox tiling: each vessel in the WSI was sliced relative to the center of the segmentation mask using a bounding box (Fig. 9b).

A total of 8212 vessels were annotated across 105 WSIs for the purpose of training DeepLabV3+. Two variants of WSI slicing into non-overlapping tiles were employed:

• Mask tiling: a tiling grid with fixed cell sizes of 2040 × 2048 and 4096 × 4096 pixels was generated for each vessel in the WSI (Fig. 9a). This grid was used for slicing the WSI.

The approach presented in Fig. 9 improves the quality of the segmentation model by providing a broader view of the input data. Vessels vary greatly in size and WSI tiling can result in situations where different fragments of the same vessel are included in the model, which can lead to incorrect results. Combining different slicing strategies, as shown in the figure, helps to model these cases in a similar way to data augmentation, allowing the model to better adapt to variations in vessel size and shape. In addition, this approach provides the model with more information about the context of the image. Different slicing strategies provide different perspectives on the same regions of the image, which helps the model to better understand the relationships between different parts of the vessels and surrounding tissues.

This approach to slicing WSIs into tiles, along with augmentations, allowed for a considerable expansion of the quantity of training data, enhanced the diversity of the visual representation of vessels, and reduced the impact of WSI tiling on the segmentation results.

A modified ComboLoss function was employed during model training to combine pixel- and region-level information.^18^ The modified loss function is represented by the following formula:

(1) $$\mathrm{Loss}=\alpha ∙\mathrm{WCE}+\beta ∙{\mathrm{DSC}}_{\mathrm{FG}}+\gamma ∙{\mathrm{DSC}}_{\mathrm{BG}}$$

where WCE — weighted cross-entropy loss, DSC_FG_ and DSC_BG_ — Dice coefficient loss for background and foreground class α, β, γ — controls the weights of corresponding loss functions (α, β, γ values are 0.5, 0.2, 0.3, respectively). Here, WCE loss allows for overcoming the data imbalance problem by giving more weight to underrepresented classes, whereas Dice loss allows for the segmentation of smaller objects. Furthermore, WCE loss provides smooth gradients, whereas Dice loss helps avoid local minima. Values of the coefficients α, β, γ were selected as a result of experiments.

ComboLoss is a composite loss function designed to leverage the strengths of both WCE and DSC. The WCE component addresses pixel-level classification, effectively handling class imbalance by assigning higher weights to underrepresented classes. The DSC components, DSCFG and DSCBG focus on region-level overlap for the foreground and background, respectively, ensuring robust performance on imbalanced datasets by emphasizing the correct segmentation of smaller structures.

This modification enhanced the model's ability to accurately segment vessels by balancing pixel-wise classification with region-level accuracy, ultimately improving the overall segmentation performance.

As a result of slicing 8212 vessels using these strategies, 14,624 vessel images were obtained. The dataset was divided into three parts (Fig. 6): training set with 10,238 images, validation set with 2193 images, and test set with 2193 images.

The Adam optimizer was used in the proposed architectures to optimize a cost function. Initial learning rate and batch size were set to 0.0001 and 16, respectively. Note that the learning rate was decreased by a factor of 0.3 at 12 epochs. The model was trained over 250 epochs, and we used an early stopping criterion to terminate the training process.

#### Classification model pipeline

For the LVI classification, three models EfficientNetB3, DenseNet121, and ResNet152 were trained. Each CNN architecture (EfficientNetB3, DenseNet121, ResNet152) had different designs and optimization strategies, resulting in differences in how they learned from the data. Each model was trained separately for 100 epochs using the Adam optimizer andWCE loss. Trained models were combined into an ensemble, which significantly improved overall performance by allowing the strengths of each model to be exploited, thereby improving the generalization ability of the system and increasing its robustness.

The rationale for utilizing an ensemble of models was based on the premise that combining multiple models can leverage the strengths of each, thereby enhancing the overall classification performance and robustness. The choice of models was particularly influenced by the scarcity of LVI data, necessitating robust architectures capable of effective learning from small datasets. Selected models demonstrated high efficiency in tissue classification tasks in WSI, as evidenced by Yang et al. and Davri et al.^19^^,^^20^ The performance of the models was evaluated using area under the receiver operating characteristic curve (AUC-ROC), F1-Score, precision, sensitivity, and specificity.

Data preparation involved the extraction of 493 tiles with LVI features and 1029 tiles of normal stroma tissue. Each image, originally 128 × 128 μm, was resized to 224 × 224 pixels for input into the models. Given the limited amount of LVI data available, the dataset was divided into training 1217 (80 %), validation 228 (15 %), and test 77 (5 %) sets.

## Results

### Segmentation–classification pipeline performance

We calculated four different evaluation metrics (FBeta, IoU, recall, AUC-ROC) to quantitatively evaluate the performance of the proposed model (Table 1).

**Table 1:** The performance of vessel segmentation models.

Model	IoU	FBeta	Recall	AUC-ROC
DeepLabV3	0.8528	0.9324	0.9209	0.9806
DeepLabV3+	0.8840	0.9553	0.9355	0.9869

Based on the results shown in Table 1, it was concluded that the DeepLabV3+ model demonstrated superior performance in vessel segmentation compared to DeepLabV3. These enhanced results were attributed to several factors related to the architecture of DeepLabV3+ and the use of a custom loss function, which was specifically designed to address the distinctive characteristics of the task. The advanced architecture and specialized loss function made DeepLabV3+ the preferred model for processing WSIs with vessels that exhibit complex morphology and significant size variability.

The results clearly demonstrated that the ensemble model achieved higher performance across most evaluated metrics compared to the individual models. With only 1522 images in the training set, of which only 493 showed signs of LVI, there was a significant imbalance in the data. Each model learned different aspects of the data distribution, leading to variability in performance metrics. For example, EfficientNetB3 had high sensitivity but relatively low specificity, suggesting that it was more prone to false positives. Conversely, DenseNet121 had high precision but lower sensitivity, indicating a stricter threshold for positive predictions.

Ensemble approach (as shown in Table 2) proved to be highly effective in the classification of LVI, offering improved accuracy and reliability over individual models. This was particularly important given the limited and imbalanced dataset, highlighting the ensemble's capability to provide more consistent and trustworthy results.

**Table 2:** The performance of the LVI classification by neural network models and their ensemble.

Model	F1-Score	Precision	Sensitivity	Specificity	AUC-ROC
EfficientNetB3	0.9565	0.9167	0.9981	0.9444	0.9949
DenseNet121	0.9524	0.9976	0.9090	0.9857	0.9899
ResNet152	0.9538	0.9736	0.9181	0.9983	0.9886
Ensemble	0.9683	0.9962	0.9878	0.9989	0.9987

### Evaluation of pathologists` work in the system

To evaluate the pathologists` work experience with the AI's assistance we created an online project within the Axon medical information system (version 1.0, Synapse Tech, Moscow, Russia) which contained three cases with WSIs of lung adenocarcinoma. Case I contained two WSIs of lung adenocarcinoma with LVI and semi-transparent masks overlaying invasion foci which were used to familiarize with the interface. Case II contained 10 WSI, of which six contained LVI foci. In this case, there were no model-generated masks. Case III also contained six WSIs with LVI features. Case III slide images were processed in the model that created semi-transparent masks indicating LVI foci.

Pathologists participating in a pilot study had been working in clinical pathology departments for at least 4 years on histological analysis, including cases of patients with lung adenocarcinoma. One doctor was from the Balashikha Hospital (Moskovskiy Region, Russia), the second was from the National Medical Research Center of Oncology named after N.N. Blokhin (Moscow, Russia). All four test tasks were conducted under video recording, which then made it possible to determine the exact duration of the assessment for each WSI. In all cases, the online system was launched via Google Chrome (version 123.0.6312.105).

**Fig. 10:**
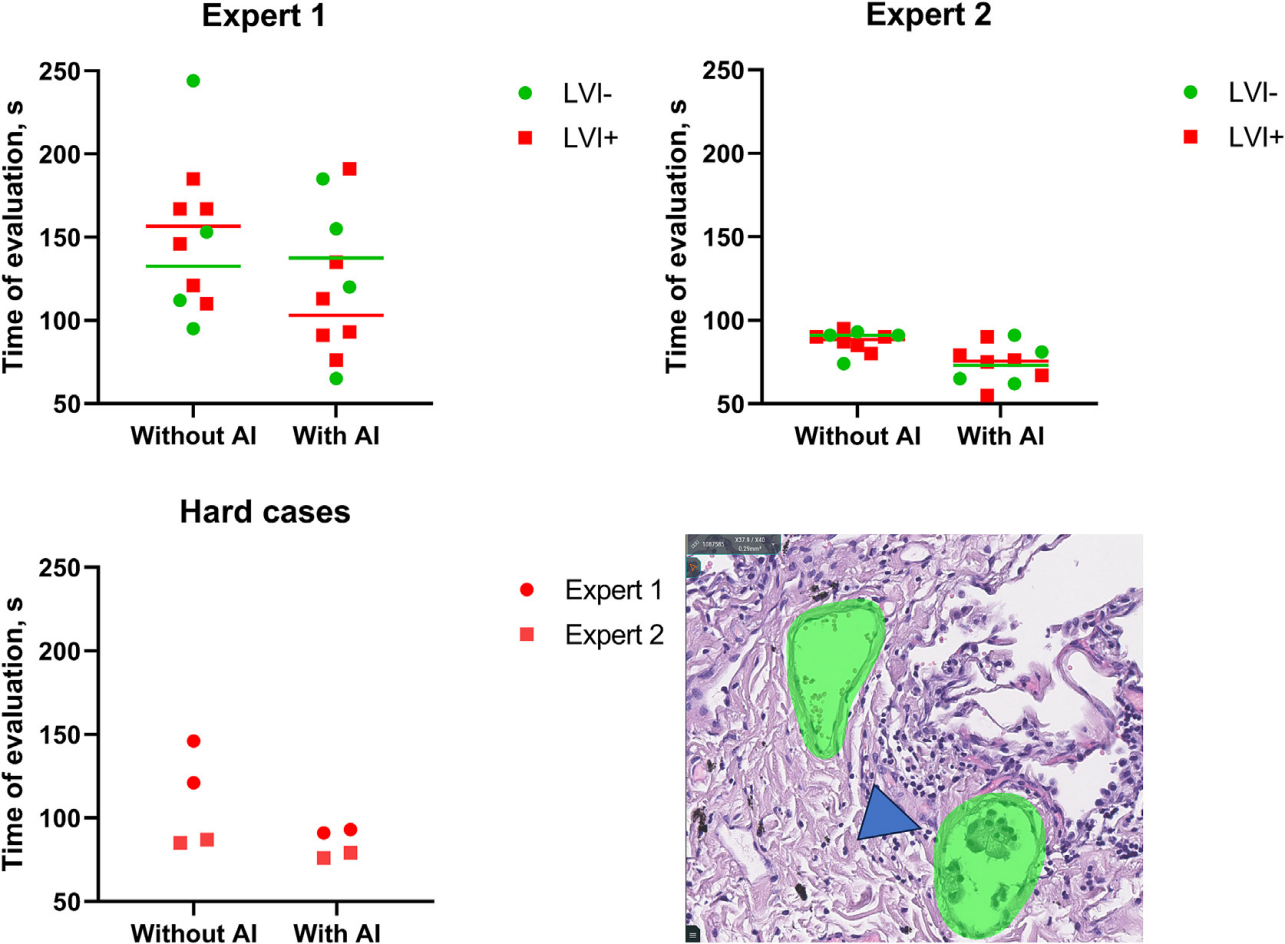
The time of evaluation of the slides by pathologists depending on the presence/absence of AI support. Experts found LVI foci faster with the presence of AI support, bars represent median values. Both experts spent less time reviewing “hard cases” with high microvascular density and singular LVI foci (arrowhead) as seen on a screenshot from an online project in the Axon system.

The average time spent by the pathologists reviewing slides was less in cases with AI support than in cases without AI support (122.4 s vs 150 s for Experts 1; 74.1 s vs 87.6 s for Expert 2) (Fig. 10). Expert 1 spent nearly twice the time that took Expert 2 to analyze the slides in cases without and with AI assistance. The time pattern of Expert 1 differed significantly primarily due to the shortening of time spent on LVI positive slides (116.5 s vs 149.3 s). Time spent on clean slide images without features of invasion did not change notably indicating that the presence of masks did not prolong the routine analysis. Expert 2 had a more moderate benefit in time spent investigating the slides, but it was observed both for positive (73.7 s vs 87.8 s) and negative (74.8 s vs 87.3 s) cases. This finding can be explained that a pathologist's work can be accelerated when supported by an AI system that he trusts.

Separately, data were visualized for “hard cases” with singular foci of LVI which posed greater risk to be missed by the pathologist. Both experts spent less time reviewing “hard cases” with singular LVI foci (92 s vs 133.5 s for Experts 1; 77.5 s vs 86 s for Expert 2). Interestingly, Expert 1 not only analyzed these cases faster but also the time spent with AI assistance was more equal.

This pilot study showed that pathologists who used the Axon information system for the first time were able to effectively identify LVI in all histological slides. The time to determine the LVI status with AI support indicated that there is a potential benefit in workload optimization (Fig. 10).

Feedback from pathologists was extremely positive, expressing a desire to implement such an AI assistant into their regular work. The pathologists noted that the neural network was excellent in identification of tumor emboli in capillaries, which in some cases can save the pathologist's time and prevent a false negative conclusion. Because such regions can be poorly defined at low magnification and the time for viewing a slide “in routine” is 70–100 s, we propose that automatic detection of such regions is the most valuable result of the model.

### Lymphovascular invasion detection in lung adenocarcinoma

**Fig. 11:**
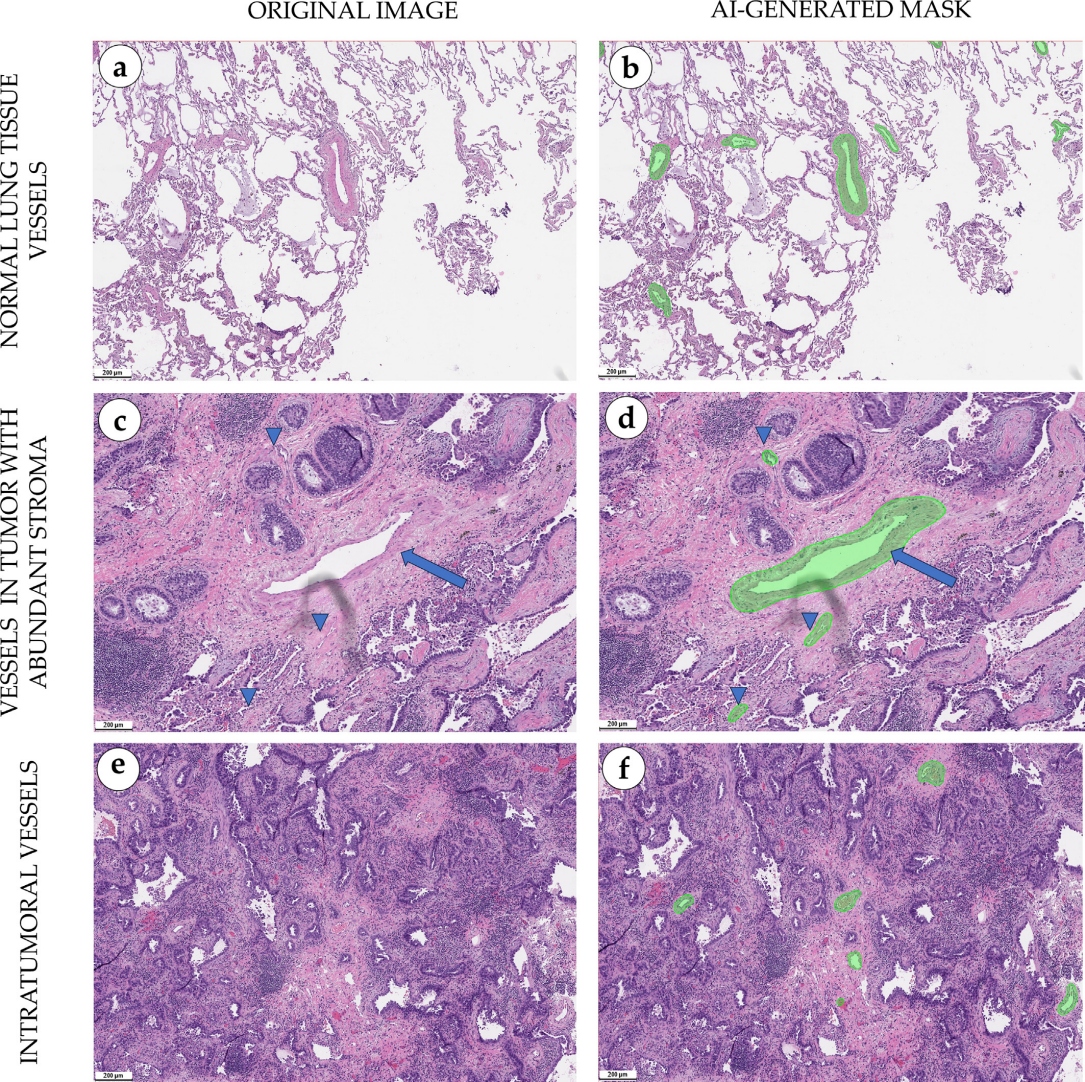
Morphological evaluation of the segmentation masks for blood vessels in lung tissue, scale bar – 200 μm. Normal lung tissue represented by a network of thin-walled alveoli lined with alveolar type I cells and containing a small amount of mucin (a). There were small foci of ciliary epithelial displacement. Intra-alveolar stroma contained singular lymphocytes and predominantly arterial vessels of medium caliber with prominent tunica media and clearly visible lumen. AI segmentation masks accurately highlighted blood vessels boundaries in normal lung tissue, including lumen and tunica adventitia fibers (b). An acinar adenocarcinoma with an abundant fibrous collagen stroma infiltrated with lymphocytes, arteria (arrow), and a net of capillaries (arrowheads) (c). Overlaying semi-transparent segmentation mask highlighted an artery (arrow) and small capillaries (arrowheads) (d). Acinar adenocarcinoma with a collagen stroma, diffusely infiltrated with lymphocytes and containing intratumoral thin-walled full-blooded capillaries (e). Segmentation mask highlighted intertumoral capillaries (e).

Vascular segmentation model generated semi-transparent masks corresponding to the contours of lymphatic and blood vessels. The model equally well identified both intra- and peritumoral vessels of different calibers (small, medium, and large). In vessels of medium and large calibers, the contours captured the fibers of the tunica adventitia. In arterioles and venules, the boundary barely went beyond the layer of smooth myocytes, because their adventitia is thin and merges with the environment. The boundary of capillaries passed closely to pericytes. Areas of branching vascular bundles with abundant stroma and large vessels with a high number of small vasa vasorum in the adventitial membrane were successfully segmented by the model (Fig. 11).

The model did not make false-positive predictions on areas of tumor stroma or components of the respiratory tract wall, including interalveolar septa and bronchioles. This was achieved because the annotation protocol strictly limited the thickness of annotated adventitial tissue, which did not exceed the combined size of tunica intima and tunica media. False-negative results of the model (absence of visualization for blood or lymphatic vessels) were minimized through the balancing of the dataset which included vessels of all calibers.

**Fig. 12:**
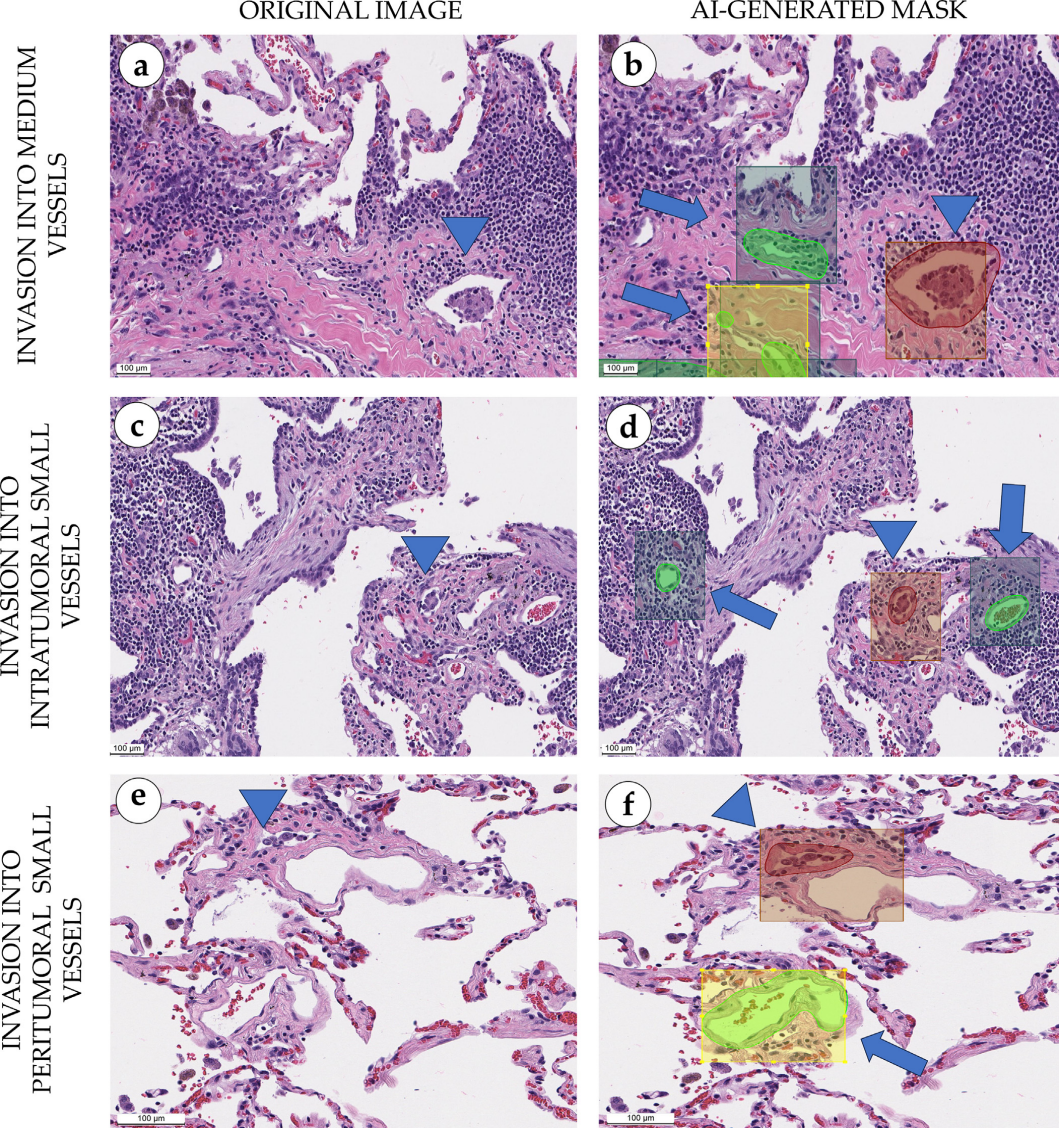
Morphological evaluation of the classification masks for adenocarcinoma cell invasion into vessels in lung tissue, scale bar – 100 μm. Normal alveoli and lung adenocarcinoma with abundant immune cell infiltration, stromal fibrosis, and a focus of adenocarcinoma invasion into a lymphatic vessel (arrowhead) (a). Semi-transparent green segmentation masks over clean (non-metastatic) vessels were overlaid by dark green/yellow classification mask which indicated absence of cancer cells (arrows); red segmentation mask highlighted lymphatic vessel with tumor emboli in its lumen and was overlaid by a semi-transparent red classification rectangle indicating an invasion focus (arrowhead) (b). Lung adenocarcinoma with abundant stromal immune cell infiltration, full-blooded vessels, and a small focus of tumor invasion into a capillary (arrowhead) (c). Green segmentation masks indicating clean capillaries were overlaid by dark-green semi-transparent classification rectangles (arrows); red segmentation mask with overlaying dark-red classification mask highlighted a capillary with adenocarcinoma emboli (arrowhead) (d). Normal alveoli with singular lymphocytes and sparse erythrocytes. Lymphatic capillary with adenocarcinoma cells in the lumen (e). Green segmentation mask with overlaying yellow classification mask over the clean vessel (arrow); red segmentation mask with overlaying dark-red classification mask (arrowhead) indicating a region of adenocarcinoma lymphatic invasion (f). (For interpretation of the references to color in this figure legend, the reader is referred to the web version of this article.)

Classification model highlighted areas of invasion with purple rectangles (Fig. 12). Tumor cells were detected in the vessel lumens and in tunicae intima and media. The model was sensitive to even singular tumor cells and effectively identified invasion within the blood vessel wall with less than a dozen tumor cells. The model proved to be particularly accurate in identifying areas of peritumoral LVI which are easier to miss than intratumoral LVI. The most valuable was the detection of invasion in small and medium caliber vessels, as they can mimic stromal clefts. The number of detected vessels with LVI features varied significantly (from 1 to 30 vessels in WSI) which allows for more quantitative characterization of the invasive nature of the tumor. Such a complex approach can present a novel prognostic biomarker when applied to a batch of histological preparations in a clinical case. Correction of boundaries and their inclusion in the final annotation protocol helped to minimize false positives, in which the model distinguished areas with the presence of inflammatory cellular infiltrate as the foci of primary tumor invasion.

## Discussion

This study presents the first model for the detection of LVI in lung adenocarcinoma. Previous research was primarily focused on vessel segmentation and microcirculatory vessel density quantification.^21^ The main challenge in vessel segmentation was the high vascular heterogeneity: each WSI contained hundreds of vessels of various calibers, ranging from large vascular branching bundles to small capillaries, including thin-walled neoformed vessels. Utilizing a segmentation model trained on an imbalanced dataset with predominance of vessels of a particular caliber led to the emergence of irregular, discontinuous segmentation masks for large vessels, and the absence of segmentation masks for a significant portion of small caliber vessels. This issue was addressed by augmenting the training process with additional annotations of microcirculatory vessel annotations.

Implementing custom loss functions, normalization, and data augmentation techniques markedly improved model generalization and effectively handled data imbalance. Nonetheless, as detailed in Boschman et al.,^14^ the benefits of normalization and augmentation diminish substantially with increase of training data. Ensembling, especially beneficial with smaller datasets, offers a considerable performance enhancement by allowing models with different architecturеs to learn various distinct features, which collectively improve the overall result. Together, these strategies increased the accuracy and reliability of our machine learning model, enabling it to perform effectively in the task with limited number of slides with invasion regions.^19^

The relatively small number of vessels with signs of LVI at the beginning of the study posed a choice: to use generative adversarial network to populate the dataset or to create a two-stage segmentation–classification model. The two-stage model proved to be effective despite its complexity. Analyzing more data will allow to balance the dataset with more vessels with signs of invasion, which will increase the computational efficiency of the model and allow to classify vessels in a one-stage pipeline.

Analysis of tumor vascular network includes both the detection and analysis of the number and size of the vessels and the assessment of the condition of their walls, the presence of inflammatory and tumor cells in them, and the finding of thrombosis in their lumen. The most advanced area of digital pathology, nephropathology, makes a great case of AI-driven transformation of routine practices for a wide array of kidney diseases.^22^, ^23^, ^24^, ^25^ In biopsies of transplanted kidneys, evaluation of the inflammation of the arteries (arteritis) is a complicated task that is crucial for appropriate clinical management.^26^ The blood vessel segmentation in kidney focuses not only on the accurate detection of blood vessels themselves but also extends to identifying specific structural components such as the endothelium. Beyond mere identification, it is crucial to measure the thickness of the vessel walls and evaluate the presence of hyalinosis, a buildup of hyaline material that can significantly impact kidney function.^27^ It was also important to adhere to strict annotation rules in our study because the presence of an abundant immune cell infiltration and a large area of connective tissue fibers in vessel wall on the training tiles contributed to an increased number of false positives for the presence of LVI foci and bronchiolar wall components. Evaluation of the presence and extent of hyalinosis in the lung may as well become useful in the differential diagnosis of sarcoidosis with tumor lesions, as well as in assessing the severity of the alveolitis.

Analyzing blood vessel patterns in cancer can help better understand the biology of the tumor. A good example is glioblastoma, which notably induces angiogenesis, leading to a highly vascularized tumor that grows rapidly and behaves aggressively. Characterized by microvascular proliferation, glioblastomas display multiple endothelial cell layers that form irregular, thickened, and distorted vessels. Assessing these blood vessels, along with other tumor features, is crucial for tumor grading, which influences treatment decisions and prognosis.^28^ AI-based techniques, particularly convolutional CNNs, have proven effective in detecting and analyzing the morphological characteristics of microvessels in glioblastoma.^29^^,^^30^ Whereas lung adenocarcinoma does not have evident vascular patterns, it is a possibility that a further morphometric analysis into blood vessel distribution inside and around the tumor can indicate its metastatic potential. Analysis of pathological vascular structures can be applied to the diagnosis of benign and malignant vascular tumors of the lung.

The presence of LVI is associated with a high risk of metastasis and poor prognosis in breast cancer. However, the morphometric attributes of LVI might not be easily observable through traditional examination. The expert–experience embedded knowledge transfer learning model identified blood and lymphatic vessels with tumor emboli in the lumen. This model further showed promising capability in predicting the likelihood of lymph node metastasis among patients confirmed to have LBVI. The most comprehensive investigations into LVI detection have been conducted in breast cancer slide images, where models for detecting invasion of breast cancer cells into lymphatic and blood vessels have been developed separately.^8^ This separate analysis of two classes of vessels can be further investigated for lung cancer as well.

Caie et al. demonstrated a computer-based methodology of LVI, tumor budding, and lymphatic vessel density quantification, which decreased observer variability. The model was employed to detect and quantify tumor blood vessels in colorectal cancer, offering insights into tumor aggressiveness, metastatic potential, and survival outcomes. A continuous hot spot probability map was developed to analyze WSIs. The colocalization of tumor budding areas with lymphatic vessels was interpreted as the invasion.^31^ The LVI-detection model can be used in the future to detect lymph node metastases and develop a prognostic model that estimates the probability of metastases depending on the presence of LVI in the lung tissue.

The main aim of the study is to establish a model for the detection of invasion, which can be missed when studying histological specimens. For additional attention of the pathologist, we intend to create a special button on the interface of the online platform, which will highlight a tile that was classified as suspicious for the presence of LVI. Better visualization of the LVI foci will increase pathologists` attention and confidence in the model's performance.

There was a number of challenges to overcome during the integration:

• The main problem was that the powerful hardware was required for the models to work quickly (Processor Intel i5/i7 12+ generation or AMD Ryzen5/Ryzen7 5000+ generation, RAM 16GB, GPU 3090).
• Everything that concerns access to medical data must be in the medical organization's internal information loop.
• The model should be integrated into officially authorized medical software.
• Integration required the additional development and update of the online platform to reflect neural network results.

Soon, digital pathology models will assist in accurate measurement of many prognostic factors, the assessment of which is highly subjective, such as microvascular density, patterns of vascularization, microvascular proliferation index, and the presence of LVI. An automated detection of these features contributes to enhancing the objectivity of histological slide analysis and opens possibilities for subsequently creating individual multi-factorial prognostic models for patients. We see our model as one of the founding bricks in a wall of data-driven framework of disease diagnostics.

Further training on other data will allow the model to be applied to other cancers where AI-measured LVI can become a histological biomarker, such as thyroid cancer and colorectal cancer.^32^, ^33^, ^34^ Leveraging transfer learning methodologies, the pre-trained model can undergo fine-tuning on fresh datasets with minimal additional training, rendering it a versatile approach for clinical translation. We believe that digital pathology researchers will find our segmentation–classification pipeline useful in analyzing vascularity of other tumors due to the extensive training incorporating images of vessels of various calibers.

A long-term study will evaluate the concordance of diagnoses between pathologists, assess the impact of the presence of LVI on patient survival and allow the revision of current oncological treatment guideline to add a specific management strategy for patients with the presence of LVI.

## Conclusions

In this study, we have demonstrated for the first time the feasibility of automated detection of LVI in lung adenocarcinoma. To train these models, a segmentation–classification pipeline was proposed effective due to its capability to detect blood and lymphatic vessels of different calibers. The experts found the model helpful and indicated that AI can change the perception of LVI from a morphological feature of interest to a histological biomarker with independent prognostic value.

The following are the supplementary data related to this article.

Supplementary Table S1 Clinical and histological metadata of WSI Supplementary Table S1

Supplementary Figure S1 Validation of the primary annotations Supplementary Figure S1

Supplementary Figure S2 Examples of distortions in the segmentation of vessel boundaries Supplementary Figure S2

## Author contributions

Conceptualization, A.T., V.A., A.F., T.D., P.T., V.M.; methodology, A.T., A.F., V.M.; software, V.A., E.Z.; validation, E.R., A.Z., N.Z., E.S., T.D.; formal analysis, E.Z., E.R. and V.Z.; investigation, A.T., A.F., E.Z., E.R., V.Z., E.S., V.M.; resources, T.D., V.M.; data curation, A.T., A.F. and V.A.; writing—original draft preparation, A.T., V.A.; writing—review and editing, A.F., A.Z., N.Z., V. Z., P.T.; visualization, A.T., V.A., A.F.,E. Z., E.S.; supervision, P.T. and V.M.; project administration, A.F., A.Z., N.Z., T.D., P.T.; funding acquisition, T.D., P.T. and V.M. All authors have read and agreed to the published version of the manuscript.

## Funding

The morphological studies were supported by the 10.13039/501100006769Russian Science Foundation (Grant No. 23-15-00481). The digital pathology studies were funded by the 10.13039/501100012190Ministry of Science and Higher Education of the Russian Federation within the framework of state support for the creation and development of World-Class Research Centers “Digital biodesign and personalized healthcare” No. 075-15-2022-306.

## Institutional Review Board Statement

Not applicable.

## Informed consent statement

We confirm that all methods were carried out in accordance with relevant guidelines and regulations. All experimental protocols were approved by Sechenov University (Moscow, Russia). Informed consent was obtained from all subjects and/or their legal guardian(s) before having their data used in this study.

## Declaration of competing interest

The authors declare the following financial interests/personal relationships which may be considered as potential competing interests:

Alexey Fayzullin reports financial support was provided by Russian Science Foundation. Vladimir Makarov reports financial support was provided by Ministry of Science and Higher Education of the Russian Federation. If there are other authors, they declare that they have no known competing financial interests or personal relationships that could have appeared to influence the work reported in this article.

## Data Availability

The data presented in this study is available on request from the corresponding author.
