# Supplementary material for: LVI-PathNet: Segmentation-classification pipeline for detection of lymphovascular invasion in whole slide images of lung adenocarcinoma
Source: J Pathol Inform. 2024 Aug 30;15:100395. doi: 10.1016/j.jpi.2024.100395 (PMC11426154; doi:10.1016/j.jpi.2024.100395)
Supplement: Supplementary Table S1 — Clinical and histological metadata of WSI [file mmc1.docx]

**Table S1.** Clinical and histological metadata of WSI

| Dataset | TNM stage | Clinical stage | Adenocarcinoma histological subtype | LVI presence | Sex | Age | Smoking status |
| --- | --- | --- | --- | --- | --- | --- | --- |
| DHMC  (143 cases) | No information | No information | 51 – solid  5 - papillary  9 -micropapillary  19 - lepidic  59 -acinar | 187 vessels | No information | No information | No information |
| NLST  (57 cases) | T2a N1 M0 – 9  T2b N1 M0 - 5  T1c N2 M0 – 23  T3 N1 M0 – 15  T2b N3 M0 – 3  T3 N2 M0 – 2 | 14 - IIb  38 - IIIa  5 - IIIb | No information | No information | 69% - male,  31% - female | 55-74 | 83% active smokers, 17% former or non-smokers |
| Sechenov University  (21 cases) | 12 - T2b N0 M0  4 - T2b N1 M0  2 - T3 N0 M0  2 - T2b N3 M0  1 - T3 N1 M1a | 12 - IIa  6 - IIb  2 - IIIb  1 - IVa | 4 - micropapillary  2 - papillary  15 - acinar | 29 vessels | 71% male, 29% female | 55-68 | 62% active smokers, 38% former or non-smokers |
