# Supplementary material for: LVI-PathNet: Segmentation-classification pipeline for detection of lymphovascular invasion in whole slide images of lung adenocarcinoma
Source: J Pathol Inform. 2024 Aug 30;15:100395. doi: 10.1016/j.jpi.2024.100395 (PMC11426154; doi:10.1016/j.jpi.2024.100395)
Supplement: Supplementary Figure S1 — Validation of the primary annotations [file mmc2.docx]

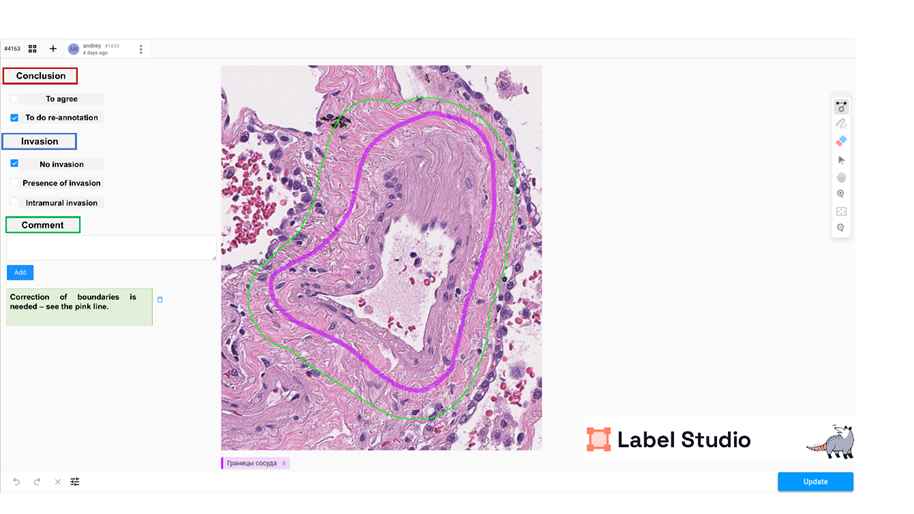
**Figure S1**. Validation of the primary annotations was performed in Label Studio. The expert evaluated each annotation separately and made a conclusion on it (red rectangle): ‘to agree’ or “to do re-annotation”. The presence or absence of invasion was evaluated as a separate feature (blue rectangle): ‘no invasion’, “presence of invasion” or “intramural invasion”. At the end of the validation, a comment was given (green rectangle) and, if necessary, the expert used the brush tool to draw new annotation boundaries (pink line) to replace the previous annotation (green line).
