# Supplementary material for: LVI-PathNet: Segmentation-classification pipeline for detection of lymphovascular invasion in whole slide images of lung adenocarcinoma
Source: J Pathol Inform. 2024 Aug 30;15:100395. doi: 10.1016/j.jpi.2024.100395 (PMC11426154; doi:10.1016/j.jpi.2024.100395)
Supplement: Supplementary Figure S2 — Examples of distortions in the segmentation of vessel boundaries [file mmc3.docx]

**
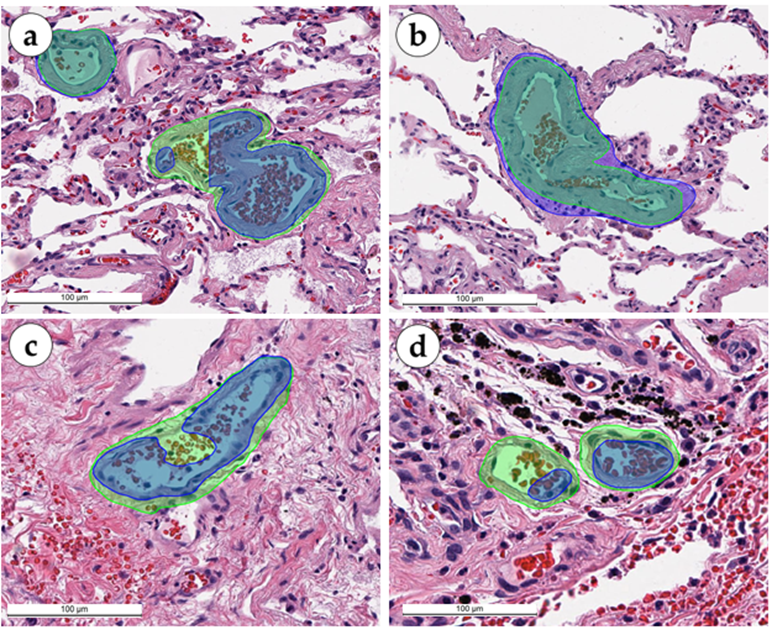
**

**Figure S2.** (a, b) Normal lung tissue with small caliber vessels, highlighted with AI-generated semi-transparent masks. (c, d) Normal lung stroma with anthracosis and full-blooded vessels, highlighted with AI-generated semi-transparent masks. Blue masks were generated without reflection application, green masks were generated with reflection application. Mask boundaries with reflection method were smoother than without reflection application. Scale bar – 100 µm.
